# How happy are your neighbours? Variation in life satisfaction among 1200 Canadian neighbourhoods and communities

**DOI:** 10.1371/journal.pone.0210091

**Published:** 2019-01-23

**Authors:** John F. Helliwell, Hugh Shiplett, Christopher P. Barrington-Leigh

**Affiliations:** 1 Vancouver School of Economics, University of British Columbia, Vancouver, British Columbia, Canada; 2 Canadian Institute for Advanced Research, Toronto, Ontario, Canada; 3 McGill University, Montréal, Québec, Canada; Mercator Research Institute on Global Commons and Climate Change gGmbH, GERMANY

## Abstract

This paper presents a new public-use dataset for community-level life satisfaction in Canada, based on more than 500,000 observations from the Canadian Community Health Surveys and the General Social Surveys. The country is divided into 1216 similarly sampled geographic regions, using natural, built, and administrative boundaries. A cross-validation exercise suggests that our choice of minimum sampling thresholds approximately maximizes the predictive power of our estimates. The resulting dataset reveals robust differences in life satisfaction between and across urban and rural communities. We compare aggregated life satisfaction data with a range of key census variables to illustrate some of the ways in which lives differ in the most and least happy communities.

## Introduction

Neighbourhoods are important places in people’s lives, both in defining the social contexts of their daily lives, but possibly also as determinants of their life chances. Children who move to better neighbourhoods have better subsequent outcomes [1] and the life satisfaction of international migrants converges to that in their new countries [2]. The key problems with estimating neighbourhood effects [3, 4], lie in separating compositional differences from contextual ones [5], and in identifying and testing possible causal pathways [6].

While there are many ways of measuring the quality of life within communities, self-reported life satisfaction has a strong claim as an encompassing umbrella measure [7]. Local, national, and global interest in life satisfaction and other measures of subjective well-being has been growing rapidly over the past twenty-five years, and is increasingly accompanied by official collection of happiness data. Of the two general types of subjective well-being measure—life evaluations, and measures of affect both positive and negative—the former is broadly considered to best capture the overall quality of life in a community or country. Thus, while the OECD has recommended a substantial slate of measures of subjective well-being [8], the slate is anchored by a core question asking people how satisfied they currently are with their lives as a whole, on a scale running from 0 to 10. While a large literature has developed analyzing the distribution and determinants of life satisfaction in cross-sectional or international contexts, it is only more recently that the collection of sufficiently large samples has allowed robust measurement at sub-national levels [9–19].

For local policymakers and urban planners interested in improving happiness in their cities, it is imperative as a first step to know where people are happy and where they are not, and also to understand in what ways the happy communities within cities differ from those which are not.

Our first objective is to meet this need by measuring the levels and distribution of life satisfaction within and across Canadian neighbourhoods and communities. The resulting dataset, and the regionalization underlying it, are also intended for use by researchers for the purposes of explaining those differences, including by providing contextual variables for analysis in combination with lower-level or individual data. By combining the data from the 2009–2014 waves of the Canadian Community Health Survey (CCHS) with the 2009–2013 waves of the General Social Survey (GSS), both of which ask the same consistently worded and scaled life satisfaction question, we create a national sample exceeding 500,000 respondents. We then agglomerate small-scale geographic units based on their natural and built geography, forming over 1200 local-level geographic entities, each of which contains a minimum of 250 survey respondents. Of these geographic entities, 776 lie within cities, and 440 in rural areas, together covering all of Canada’s geography.

The community-level means are tightly estimated in our data, with standard errors only about 1% of the mean. Community level averages range from 7.0 to 8.9, more than twenty standard errors. Life satisfaction levels differ substantially across the neighbourhoods within large cities, with a range substantially greater than that which has previously been observed across cities themselves [13]. Meanwhile, life satisfaction in towns and rural areas is generally higher than in cities, with less variation across communities, though outliers are present. The happiest and least happy urban neighbourhoods differ, significantly, across almost all of the social, economic, and demographic dimensions which we consider. On the other hand, the happiest and least happy towns and rural areas only show significant differences in religiosity, inequality of well-being, sense of community belonging, housing affordability, and length of residential tenure, and do not differ significantly along other measured dimensions, including income, unemployment, and education.

Nationally, the happiest and least happy communities differ markedly in their residents’ sense of community belonging, population density, inequality of well-being, and time in residence, and less so in income, unemployment, and education.

## Background

High geographic resolution in accounts of well-being are of value to researchers and policy makers alike as the drivers and supports of well-being have strong local components [20]. Trust in neighbours and sense of belonging to one's local community, for instance, predict life satisfaction beyond their influence on other measured community and individual characteristics [21]. Potentially salient local spillovers such as these can only be effectively studied if measures are available at both the individual and higher contextual levels.

These spillovers, along with social norm- and reference-setting, are also likely to operate differently at large and small geographic scales. In the extensively studied area of income reference effects, for example, results have differed depending on the physical and social proximity of the population to whom comparisons are made [22–24].

Numerous other social, economic, and demographic determinants including ethnicity, housing type and housing costs, access to services, and so on all vary locally and have natural implications for life satisfaction [13, 25, 26]. Studies which average spatially over all these sources of variation will tend to underestimate their importance. This lack of variation, combined with the resulting drop in the number of communities under study, renders it difficult or impossible to identify the underlying relationships.

The usefulness of high-resolution life satisfaction datasets is also complemented by the availability of compatibly geo-coded data. The smallest geographic scales at which census data are compiled represent natural building blocks for analysing life satisfaction, as there is a wealth of spatial analytic data from government and other sources that can be brought to bear on the task of understanding the determinants of life satisfaction.

On the other hand, life satisfaction is particularly challenging to measure at small geographic scales. It has a large idiosyncratic component at the individual level, manifested as unexplained variance in most modeling efforts. As a result, for reasons of cost, there are relatively few datasets available with local sampling. National surveys tend anyway to stratify at larger spatial scales, and very large samples must be accumulated in order to have both full coverage and the ability to statistically discriminate at fine spatial scales. Nonetheless, as sample sizes have increased, efforts to illustrate the spatial distribution and predictors of happiness within nations have been undertaken at increasingly fine geographic resolutions, including at the level of provinces in Europe [9, 16], US states [17–19] and subsequently counties [11, 12], and cities in the United States [10] Canada [13] and New Zealand [14], among others.

The Canadian context is unusual in this regard, as the combination of large survey samples and a relatively small population have resulted in exceptionally high sampling densities. The half million observations of life satisfaction in the Canadian CCHS and GSS samples used in this study constitute nearly a two percent sample of the adult population. This allows us to describe life satisfaction across Canadian neighbourhoods and communities with an unprecedented level of geographic granularity.

## Methodology

### Theoretical considerations

A previous geographic breakdown of Canadian life satisfaction data [13] included fewer than 100 geographic units, with each metropolitan area (CMA) treated as a single unit. This led to large variations in sample counts among communities, but it did reveal that life satisfaction in general is lower in the large cities than in less densely populated parts of the country [13]. Much of the increase in the number of communities which we provide, from 98 to 1216, has come from delineating up to dozens of neighbourhoods with roughly equal sample sizes within each CMA.

Although the combined CCHS and GSS samples are large enough to permit this community-level geographic disaggregation, official geographic units suitable for reporting these statistics are not currently available. The largest sub-municipal units, census tracts, have an average sample size of 68, and remain unevenly sampled by both surveys, which are stratified at much higher levels such that over 50% of census tracts have sample sizes below 50, and approximately 20% have sample sizes below 25. As a result, it was necessary to delineate intermediate-level statistical reporting regions suitable for our purpose. The choice of both the scale and boundaries of these units, however, is non-trivial and it is well known that these selections give rise to the modifiable areal unit problem (MAUP), and can have large and complex effects on the patterns and relationships in the resulting data [27–33].

In light of these considerations, our implementation is guided by three general principles that affect its quality both theoretically and practically:

*(1) A trade-off exists between geographical and statistical precision*.

Simply put, our geographic units should be large enough to provide usable sample sizes, yet small enough not to obscure the underlying patterns by combining overly dissimilar communities, paving over their differences. In the language of the MAUP, this is commonly referred to as the ‘scale effect’. While our initial choice of scale was heuristic in nature, the results of a subsequent cross-validation exercise, described below, support our selection.

Even once an appealing spatial scale has been chosen there are many potential regionalizations consistent with it, and some of these may be better than others. Consider even the extreme, and unrealistic case, in which it could be determined with certainty that all neighbourhoods were perfect squares exactly *x* miles across; the correct alignment of this grid would remain to be determined. While all of a person’s neighbours would live within *x* miles of them, not everyone within *x* miles of them would their neighbour. This motivates our second objective.

*(2) Conditional on their geographic scale*, *boundaries should be drawn such that they combine populations which are likely to be socially connected and separate those which are not*.

That the positioning of the boundaries in a given geographic partition can affect the patterns displayed by the resulting data, the ‘zonation effect’, should be familiar to many readers due to the oft-noted problem of “gerrymandering”. For example, electoral boundaries are sometimes redrawn with the aim of improving outcomes for the governing party (gerrymandering), and subsequent results are often attributed to the boundary changes [34]. However, one recent study [35] in the US context has shown the importance of separating the electoral effects of boundary changes from what would have happened anyway as a consequence of underlying changes in the population mix in those same areas. In our context, we wish to minimize such distortions in our description of variation across local communities, which we assume to be at least partially geographically defined, and thus attempt to draw our boundaries around them rather than through them.

Our approach to this, described in more detail below, has been to make use of pre-existing boundaries at both higher and lower levels which have been determined with similar motivations, and also to make heuristic use of natural and built geography.

The third principle is largely practical.

*(3) As much as possible*, *new geographic units should be compatible with the major pre-existing higher- and lower-level delineations*.

Our boundaries are designed to be consistent with those of the various nested geographies employed in the census. This facilitates aggregation of demographic and economic variables to the same neighbourhoods, as well as to broader regions in which they are contained. This objective might be seen as conflicting with (2); in practice, these objectives are complementary, since the census tract boundaries are chosen using criteria similar to ours.

### Implementation

We formed agglomerations of small-scale census geographical units with known sample sizes by using natural and built geography and higher-level statistical boundaries to guide the delineation of regions. This was accomplished using sample sizes for life satisfaction variables from the 2009–2014 CCHS and the 2009–2013 GSS surveys for all census tracts (CTs) and census subdivisions (CSDs) in Canada.

Census subdivisions (CSDs) correspond to municipal or similar administrative boundaries. These administrative boundaries are determined at the territorial or provincial level, are non-overlapping, and cover the entirety of Canada. Due to variation in provincial and territorial delineation practices, as well as substantial variability in the size of Canadian municipalities, CSDs are not of consistent size.

Consequently, wherever possible, CTs were used as the basis for our aggregate regions. CTs are small, relatively uniform and stable geographic units defined only within Census Metropolitan Areas (CMAs) and Census Agglomerations (CAs) with core populations of 50 000 or more. Wherever they exist, CTs were used as base units for our aggregation instead of CSDs for several reasons. First, their small size and uniformity, with populations generally ranging between 2500 and 8000, corresponding to sample sizes averaging approximately 50, allowed us greater flexibility in choosing boundaries for aggregate regions, generally comprising several CTs. Second, CTs are delineated by committees of local experts in cooperation with Statistics Canada, and must correspond to known permanent physical features. Using CTs wherever possible thus allowed us to leverage information about relevant community boundaries more effectively than would have been feasible if we had started from finer geographic units such as dissemination areas. Since CTs perfectly subdivide CMAs and CAs, which are themselves composed of CSDs, no overlap occurred between aggregate regions composed of CTs or CSDs, and full coverage of Canadian geography was maintained.

The CCHS and GSS are both broadly representative of the Canadian population, but their sampling frames do have some limited exclusions. The CCHS excludes individuals residing in institutions (e.g. prisons, assisted living facilities, military bases), as well as on reserves or in other indigenous settlements. After these exclusions, the CCHS still covers over 97 percent of the Canadian population aged 12 and over. Similarly, the GSS is restricted to individuals in private households aged 15 and over. As a result, although our aggregate regions cover all of Canada’s geography, they do exclude a small proportion of individuals not sampled in the CCHS and GSS, which may comprise substantial proportions of the underlying populations in a limited number of cases, particularly when an aggregate region contains or overlaps with a large military base or a reservation.

Sample counts for each of 5401 CTs and 4207 CSDs from the combined CCHS/GSS life satisfaction sample were linked to 2011 census boundary files using ArcGIS. A target cell count range of 300 to 500 was initially selected for the new regions, which was achieved by combining CTs and CSDs by hand, according to the process outlined below. In order to accommodate cases where achieving a cell count above 300 would generate an implausible region or require a region to straddle higher-level statistical boundaries, cell counts between 250 and 300 were tolerated. Similarly, regions with cell counts higher than 500 were tolerated if they were deemed to match underlying features well.

Since the CCHS and GSS samples are stratified at much coarser levels than the CT or CSD, sample counts in higher resolution geographic units are not perfectly proportional to their populations. Therefore, to ensure data quality, we use a sampling criterion as opposed to a population criterion. In the case of life satisfaction, the individual idiosyncratic component of variance turned out to be large relative to the variance across geographic areas. Thus, since most of the variation is within rather than among units, targeting uniformity in sample sizes allowed us to approximately minimize the average sampling error for our units. Regions outside tracted CMAs had the same cell count rules as those within, excepting that since non-tracted CSDs could not be broken down further, resulting in 23 regions with cell counts above 1000.

The decision to proceed manually as opposed to using an agglomeration algorithm was due to several factors. First, due to the high degree of variation within as opposed to between CTs, the measurement error on their mean values is large relative to the total variation between CTs. This problem could be exacerbated by algorithms designed to induce homogeneity in life satisfaction within agglomerations by pairing CTs in part on the basis of these errors. Second, with the potential effects of the MAUP in mind, an algorithm favouring internal homogeneity in some set of potential explanatory variables could generate regions tending to favour these variables over others in subsequent analysis by generating units which differentially conform to the scale and distribution of the processes in which they are involved. Alternatively, using patterns in road connectivity allowed us to pursue a criterion which can be understood directly as both a powerful influence on and a reflection of the physical structure of our communities [33], and which we feel is otherwise plausibly neutral. Though laborious, a manual approach was deemed feasible, and given the fundamental role of pattern detection in this approach, was preferred.

Within tracted CMAs, census tracts with their sample counts were overlain with road maps and other census boundaries in ArcGIS. Agglomerations of CTs were designed to be compact and to encompass areas that were well-connected internally by the underlying road structure. Natural and built barriers such as rivers, highways, and railroads as well as other breaks in road connectivity or abrupt changes in building patterns served as boundaries wherever possible. Additionally, in order to leverage the quality of information in CT boundaries, in cases where multiple 2011 CTs had been split from a single 2006 CT, it was preferred to recombine them within this original CT. Wherever possible, CSD boundaries were followed in order to maximize compatibility with the broad range of data-sets which use census geography. Since census tracts are initially delineated by committees of local specialists, re-combining census tracts that were split in 2011 or previous census years was strongly preferred in order to take advantage of this additional information.

Outside tracted areas, CSDs were combined using natural features as well as CMA/CA, Economic Region (ER), and Census Division (CD) boundaries. Given the rigorous and more consistent delineation of CMAs, CAs, and ERs by Statistics Canada, conformability with these boundaries was given a higher priority than with CD boundaries, the delineations of which are less consistent across provinces. Likewise, since ERs are composed of CDs, in the very small number of cases where CMA/CA and ER boundaries conflicted, the former received priority. Although the creation of disjoint regions was strongly avoided, there were a few cases where CMA/CA and ER boundaries produced isolated areas without sufficient counts to become aggregate regions. In such cases, the isolated areas were combined with nearby regions, generally from the same CD. The only cases where CMA/CA boundaries were not followed were when the CMA/CA did not contain a sufficient number of observations, in which case outlying CSDs were added. Due to the nature of the aggregation procedure, in which all regions are composed exclusively either of CTs or CSDs, all boundaries of tracted CMAs and CAs are followed. Similarly, no aggregate regions overlap provincial or territorial boundaries.

### Validation

As discussed above, there are trade-offs implicit in the selection of any aggregation scale. While the scale of aggregation was initially chosen heuristically, we undertake a cross-validation exercise which provides a data-driven way of evaluating this decision.

We start by splitting census tracts into two sub-samples by randomly selecting one census tract from each of our hand-built regions. The selected ‘validation’ CTs are omitted from the main data-set and placed into a validation sample, with the remaining census tracts placed in the ‘estimation’ sample.

For any given partition of the sample, *J*, induced by a sampling threshold *n* which yields *N*^*J*^ regions, we can then use the mean life satisfaction among the estimation census tracts in each region to predict the mean life satisfaction in the region’s omitted census tract. This is accomplished by OLS regression using the following specification

$${LS}_{ij}=\beta_{0}+\beta_{1}{\bar{LS}}_{j}^{-i}+u_{ij}$$

where *LS*_*ij*_ is the mean life satisfaction in omitted tract *i*, located in region *j*∈*J*, and where

$${\bar{LS}}_{j}^{-i}=\frac{1}{\sum_{(k\neq i)\in j}w_{kj}}\sum_{(k\neq i)\in j}w_{kj}{LS}_{kj}$$

where *w*_*kj*_ is the sum of weights in tract *k*, so that ${\bar{LS}}_{j}^{-i}$, gives the survey-weighted average life satisfaction among the estimation tracts in the omitted tract’s region. In practice, since census tracts are unevenly sampled, the observations on the left-hand side variable are measured with different levels of noise across observations. To account for this, the observations in this regression are weighted by the sum of weights in the omitted census tract.

We can then compare any two regionalizations *J* and *J*′, which use different sampling thresholds, by comparing the fit of the regression of life satisfaction in the omitted tracts on the leave-out means induced by *J* and *J*′. Intuitively, when *n* is small so that *N*^*J*^ is large, meaning that the regions being used to predict *LS*_*ij*_ are small, ${\bar{LS}}_{j}^{-i}$ uses a small number of observations sampled nearby. Thus it provides a noisy but unbiased estimate of *LS*_*ij*_. Conversely, when *n* is large so that *N*^*J*^ is small, ${\bar{LS}}_{j}^{-i}$ uses a larger number of observations which are on average farther away, providing an estimate with lower variance, but which is biased in the direction of the average in the broader region. The optimal scale will balance these two effects to achieve the minimum mean squared prediction error.

When the omitted tracts are held constant, maximizing the R-squared from these regressions is the same as minimizing the mean squared error. In the results below we will use the regression R-squared, as it allows us to compare relative predictive success and also has an intuitive interpretation in this univariate setting as the square of the correlation between *LS*_*ij*_ and ${\bar{LS}}_{j}^{-i}$.

Implementing the procedure outlined above, however, requires us to generate a large number of alternative regionalizations. To do so, we implemented a simple regionalization algorithm designed to simulate the use of a sampling threshold in generating compact, contiguous regions, but ignoring the physical and built geography.

Our algorithm starts by collapsing census tracts to their centroids, which are allocated to the cells of a very coarse latitude/longitude grid. The algorithm then proceeds to split any cell of the grid which contains more than the user-specified threshold number of observations by bisecting it along its shortest axis. If the split results in both sub-cells falling below the threshold, the split is undone, otherwise the algorithm continues. When no more splits are possible, any cells with sample sizes below the threshold which have been created as a result of being split from larger cells, are merged with their neighbours until all cells meet the sampling criterion. The resulting regions tend to be compact, due to the nature of the grid, and are approximately contiguous. Since the tracts are assigned based on the proximity of their centroids with no explicit contiguity requirement, sometimes water features or irregularly shaped neighbours can bisect the algorithm’s regions. To increase precision, the procedure is repeated for multiple draws of validation tracts, as well as with different randomly assigned offsets to the starting grid.

It is important to note that the optimality of a scale in this case is conditional on the zonation methodology used to draw the boundaries themselves. In this case, we will be using a procedure designed broadly to mimic our own, in that it will favour compactness and contiguity, but which does not take advantage of physical and built geography, as we have tried to. To the extent that the procedures are different, the performance of the algorithmically generated regions at different scales provide only a rough guide to the trade-off implicit in our own choice of sampling thresholds. Furthermore, given that we used more information in our regionalization than the algorithm, we hope to find that our method outperforms the algorithm. Thus, an ideal result would be to find that the R-squared from using our regions is above that obtained from using similar sized regions generated by the algorithm, and that the sample size in our regions is near that at which the R-squared from the algorithm’s alternative regional splits reaches its maximum.

**Fig 1:**
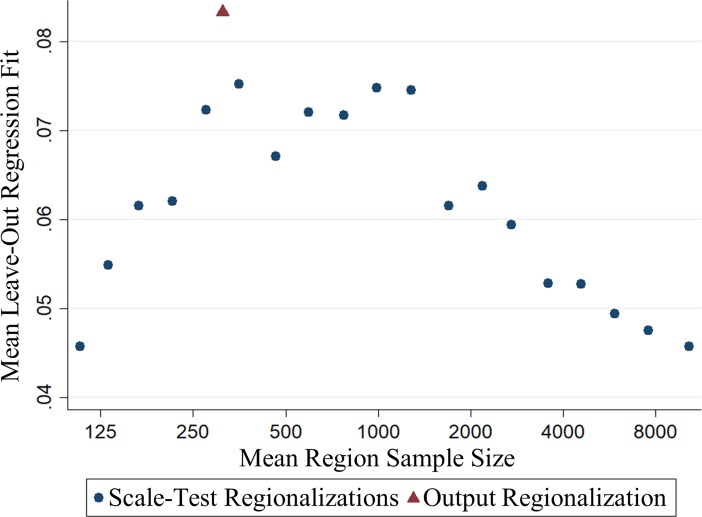
Goodness-of-fit from regressing mean life satisfaction in validation CTs on mean life satisfaction in estimation CTs using regionalizations of varying geographical coarseness.

This is indeed what we find, as shown in Fig 1, which shows the average R-squared obtained from regressing mean life satisfaction in the validation CTs on mean life satisfaction among the remaining CTs in the region to which they are assigned, for a range of sampling targets. The horizontal axis gives the average sample size per region for each target, which ranges from 50, the size of a typical CT, to over 8,000, which would correspond to a small CA or CMA. As expected, as we move from smaller to larger regions, our ability to predict life satisfaction in the validation CTs rises as long as the effect of larger samples dominates. At average sample sizes above 500, the detrimental effect of smoothing over local variation begins to dominate the limited gains from additional observations, and the predictive power starts to fall again. In the optimal scale range, which appears to lie between regional samples averaging between 300 and 1000, the regional means are able to explain over 60% more of the variance in life satisfaction across validation CTs than regional groupings at either extreme of the scale range.

We are pleased to see that our method, given by the red triangle in Fig 1, lies above the line of the algorithmically generated regions, and explains a significantly higher fraction of the total variance across CTs than is obtained from any of the comparator procedures, and nearly twice as much as those at either extreme of the scale range. Even more importantly, our chosen sample size approximates very closely the sweet spot implicit in the results from the test procedures–the place where the gains in precision in the estimates of the sample means are offset by losses in the relevance to local conditions.

## Results

### Describing the data

**Fig 2:**
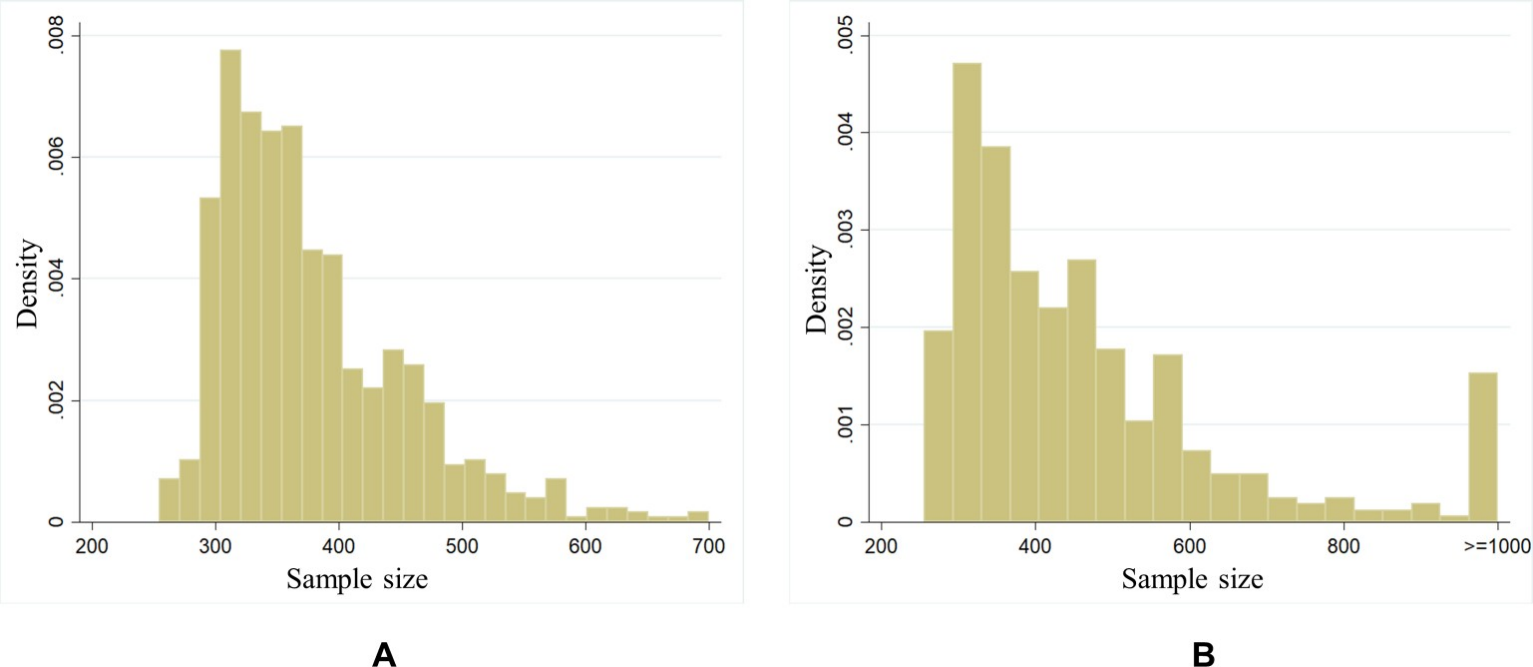
Distribution of life satisfaction sample sizes. (A) Tracted regions, located in CMAs and CAs with core populations of 50 000 or more. (B) Non-tracted regions, located outside of tracted urban centres.

In all, 1216 regions were created, spanning the entire country, of which 776 are located in tracted CMAs and CAs. Among these, 86% of the new aggregate units have cell counts within the target range of 300 to 500, with only 48 having cell counts between 250 and 300, and none below 250. Similarly, only 38 of the 440 non-tracted regions fall in the 250–300 range, with all others at 300+. Fig 2 plots the distribution of sample sizes for non-tracted and tracted regions, respectively.

**Fig 3:**
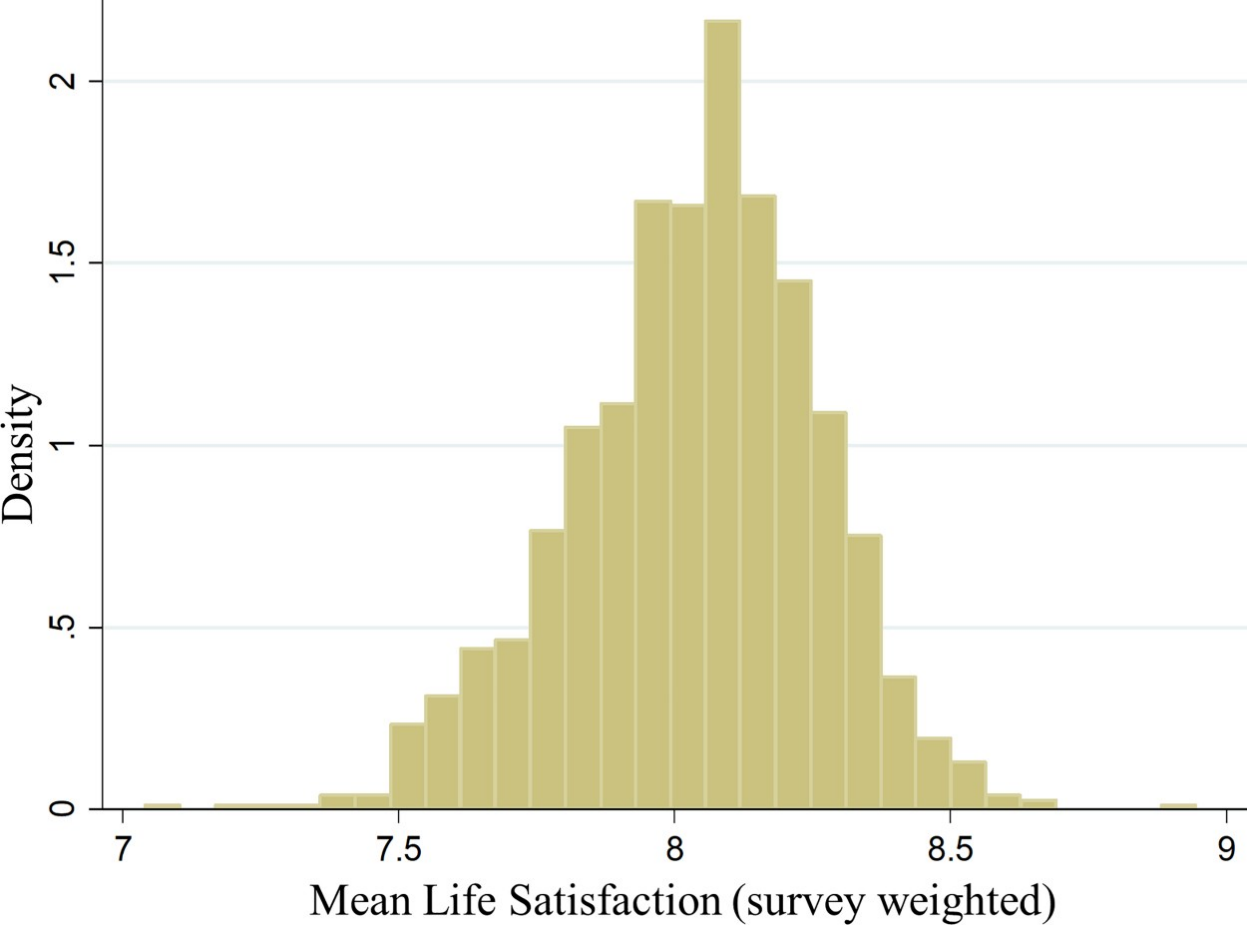
The distribution of mean life satisfaction across regions on an 11-point Likert scale.

The distribution of mean life satisfaction across regions is shown in Fig 3. We find substantial variation in mean levels of life satisfaction across regions, with a range of 7.04 to 8.96 and a standard deviation of 0.22. Even when outliers are eliminated, the range remains over one point on the 11-scale. Based on the calculated standard errors, which average 0.08, 337 of the 776 urban were significantly different from the urban mean at p < 0.05. The variation across communities within cities is larger than that between cities. For example, the range of mean life satisfaction within Canada’s three largest cities of Toronto, Montreal, and Vancouver were 0.97, 0.98 and 1.21, respectively. This is approximately twice the range across Canada’s CMAs and ERs [13].

**Fig 4:**
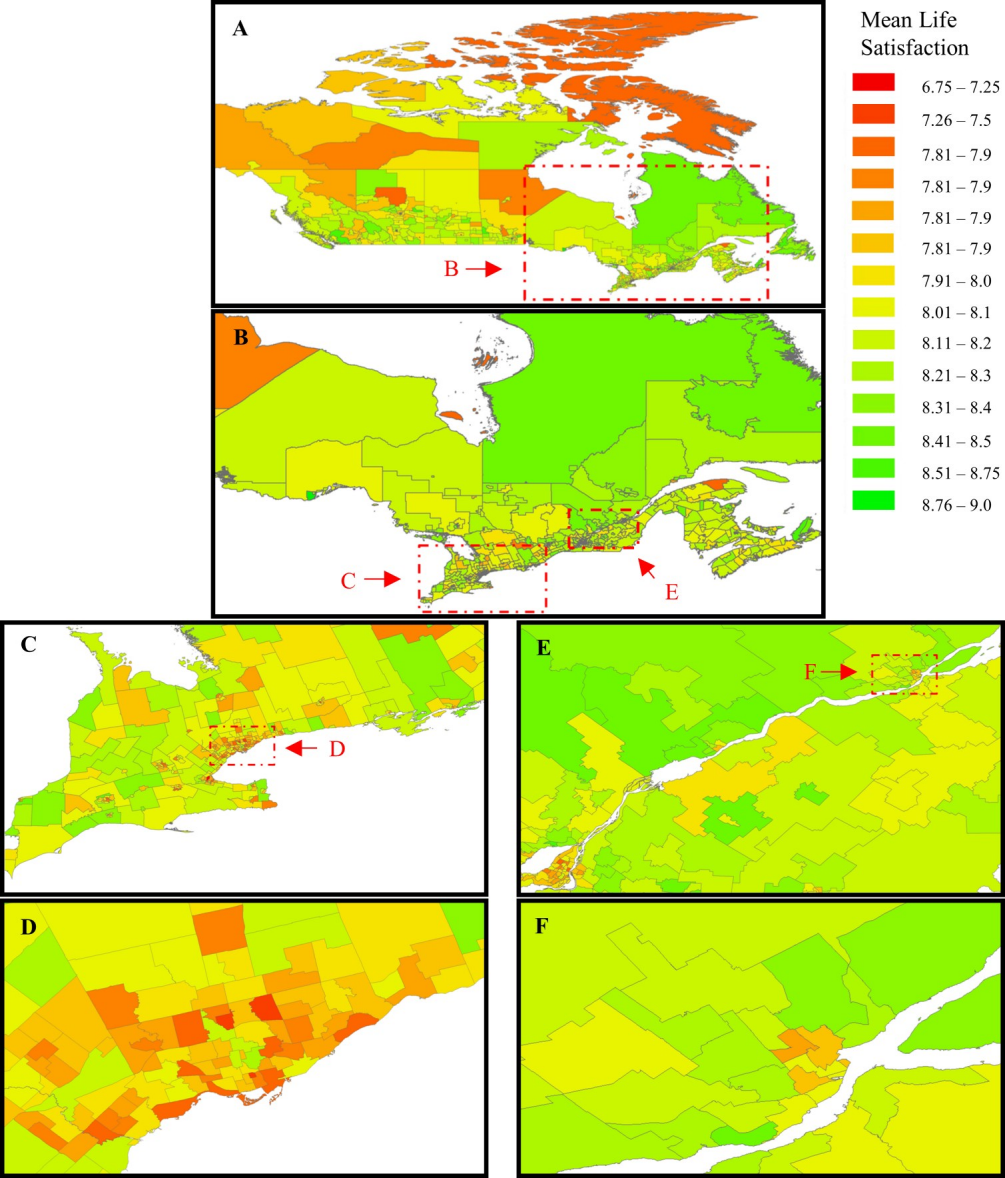
The geographic distribution of mean life satisfaction across Canada.

The panels of Fig 4 also provide a visual introduction to the data, starting with the country as a whole, narrow to the Toronto-Montreal-Quebec City corridor, and then to Quebec City and its environs. Already there is some hint that big cities are not happy havens, even if Quebec City is the happiest among them [13]. The high relative happiness of the Province of Quebec and of Quebec City is the consequence of a remarkable 25-year upward trajectory of life satisfaction in Quebec relative to the rest of the country [36].

Table 1 provides descriptive statistics for average life satisfaction and its within-community standard deviation, as well as the sense of belonging to the local community, in addition to demographic and economic characteristics commonly studied in the literature. While life satisfaction, and community belonging are taken from the combined CCHS/GSS sample, all other variables are taken from the 2011 National Household Survey (NHS), aggregated to the same communities. The first three variables in Table 1 are the only ones drawn from surveys. All of the rest are based on census averages for the matching geographic units. The NHS variables include mean household income, the unemployment rate, the average commute duration, and the log of population density, as well as the proportions of individuals who are foreign born, identify with a religion, identify as indigenous, have resided at the same address for more than 5 years, have completed tertiary education, and who spend more than 30% of household income on housing, a crude but straightforward measure of housing affordability.

**Table 1:** Summary statistics.

Variable	Mean	Std. Dev.
Satisfaction with Life	8.04	0.23
Std. Deviation of SWL	1.66	0.19
Community Belonging	0.73	0.08
Log Mean HH Income	11.23	0.27
Unemployment (percent)	7.95	3.81
Housing Over 30% of Income	0.23	0.08
Proportion Foreign Born	0.17	0.16
Proportion Religious	0.76	0.13
Proportion Indigenous ID	0.06	0.12
Proportion Resided 5+ Years	0.62	0.10
Proportion University Degree	0.62	0.10
Median Commute (minutes)	19.12	6.69
Log Population Density	5.24	2.88

### How happy are they, and are they different?

We now return to the question posed in the introduction.

*How happy are the happiest communities relative to the least happy*, *and how do these differences compare with the average values for other variables?*

As discussed above, the answer to this question is of importance for two reasons. In the first place, knowing which neighbourhoods and communities are happy places and which are not is of first order importance to decision makers who value subjective well-being as an outcome of policy, just as are local accounts of key economic indicators. Second, establishing empirical regularities provides a foundation for subsequent research on how to build and support flourishing communities. While it can be misleading to think in causal terms, simple cross-sectional relationships, both expected and unexpected, can highlight and motivate fruitful avenues of inquiry.

**Fig 5:**
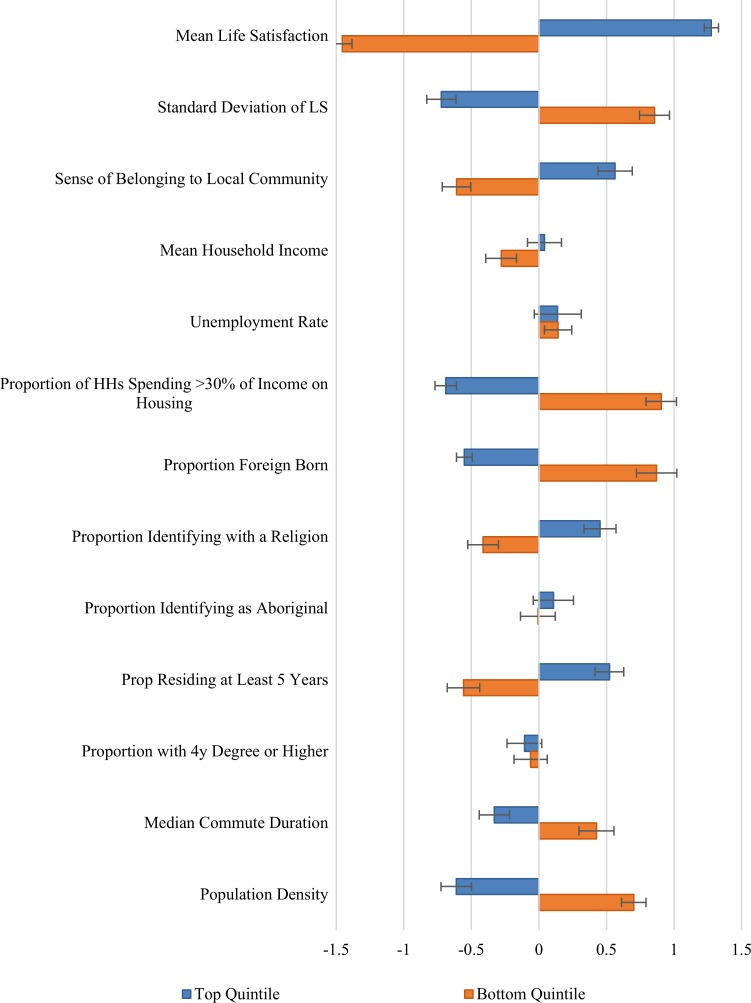
Difference of top and bottom quintile means from the mean region. Error bars represent heteroscedasticity robust 95% confidence intervals. Values for each variable are normalized to the standard deviations given in Table 1.

In keeping with the spirit of this question, and with the caveat that determining causal relationships lies beyond the scope of the present study, we provide a direct comparison of the happiest and least happy communities, namely those in the top and bottom quintiles of the distribution of life satisfaction. In Fig 5, we show the amount by which the top and bottom quintile averages for each variable differ from those of the 1216 communities taken together, with error bars indicating 95 percent confidence intervals. The differences are made more comparable by being normalized so that the unit of measure is the standard deviation of the variable in question. The raw means and differences are also presented in Table 2. It is important to note that Fig 5 shows the size and significance of inter-quintile differences one variable at a time. Many of the variables are correlated with one another, for example population density, the share of the population that is foreign born, and the proportion of families spending more than 30% of their household incomes on housing are all much higher in urban than rural areas.

**Table 2:** Differences between means of top and bottom life satisfaction quintiles.

Variable	Top Quintile	Bottom Quintile	Difference
Satisfaction with Life	8.33 (0.01)	7.70 (0.01)	0.63 (0.01)
Std. Deviation of SWL	1.52 (0.01)	1.82 (0.01)	-0.30 (0.02)
Community Belonging	0.77 (0.01)	0.67 (0.00)	0.10 (0.01)
Log Mean HH Income	11.24 (0.02)	11.16 (0.02)	0.08 (0.02)
Unemployment (percent)	8.26 (0.36)	8.91 (0.20)	-0.65 (0.41)
Housing Over 30% of Income	0.17 (0.00)	0.30 (0.00)	-0.13 (0.01)
Proportion Foreign Born	0.08 (0.00)	0.30 (0.01)	-0.23 (0.01)
Proportion Religious	0.82 (0.01)	0.71 (0.01)	0.11 (0.01)
Proportion Indigenous ID	0.05 (0.01)	0.06 (0.01)	-0.01 (0.01)
Proportion Resided 5+ Years	0.68 (0.01)	0.57 (0.01)	0.11 (0.01)
Proportion University Degree	0.61 (0.01)	0.62 (0.01)	-0.01 (0.01)
Median Commute (minutes)	17.01 (0.39)	21.82 (0.45)	-4.81 (0.60)
Log Population Density	3.47 (0.17)	7.15 (0.16)	-3.68 (0.23)

Heteroskedasticity robust standard errors are in parentheses.

There are large differences in average life satisfaction between the top and bottom quintiles, from an average of 7.7 in the least happy quintile to 8.33 in the top quintile. Since the life satisfaction means are measured quite precisely–with a standard error of about 0.08 –the differences among communities are highly significant. The inequality measures also differ, with the distribution of life satisfaction being significantly more equal in the happiest quintile. There are also large and highly significant differences in the sense of community belonging. Earlier research [20] has shown that several measures of trust help significantly to explain differences in life satisfaction across communities and nations. Only the GSS has measures of local and general trust, so the sample sizes are too small to be meaningful for our 1216 communities. A sense of community belonging, which is measured in both the GSS and CCHS, can be seen as a partial proxy for neighbourhood-level trust measures, since it has previously been shown in the GSS data to be correlated with measures of neighbourhood trust, and to be an even stronger predictor of life satisfaction [21].

Neither household incomes nor unemployment rates differ significantly between the top and bottom quintiles. This may to some extent be just another way of looking at the rural/urban happiness divide, as incomes are lower and unemployment rates higher in the rural communities. Individual-level life satisfaction data show significant positive effects from household income and negative effects from unemployment, and the same is also true when we come to compare the most and least happy quintiles of the urban distributions, although not for the rural sample or, as we see here, for the entire national sample.

The top and bottom quintiles do differ significantly for the first three of the population proportion variables: those spending more than 30% of their household income on housing, the proportion of the population that is foreign born, and the proportion who identify with a religion. By contrast, the indigenous population shares are identical in the most and least happy communities. In both quintiles the indigenous population shares average about 6%. The range of indigenous population shares is very large, and equally so in both happiness quintiles, with community average indigenous population shares ranging from 0 to over 90% in each.

The proportion of the population residing 5 years or more is significantly higher in the happiest quintile, while the population share with tertiary education is equal in both quintiles. Median commuting times and population density are significantly lower in the happiest communities, while unemployment rates do not differ between top and bottom quintiles. Commuting times average 17 minutes in the top quintile, and five minutes longer in the bottom quintile, a statistically significant difference. By contrast, population density in the least happy quintile is more than eight times greater than in the happiest quintile. This latter finding is consistent with previous research in several countries, including Canada [13], the United States [10] and Denmark [37], showing that life is significantly less happy in urban areas. We now split the data accordingly to address this difference.

### The urban/rural gap

It is already apparent from previous findings that big city life is less happy, with two of Canada’s biggest cities, Vancouver and Toronto, in a virtual tie for bottom spot among all 98 CMAs and Economic Regions [13]. Yet migrants generally, and immigrants especially, choose to move to larger cities. These moves may be driven by employment [10] and family reasons., while migrants may be unaware of either the nature or the reasons for average life satisfaction being lower in the large cities. For most foreign migrants to Canada’s large cities, life is in any event far happier than in their source countries [2].

We observe a gap in life satisfaction when dividing our sample into urban (tracted) and rural (non-tracted) samples as well. The tracted regions, located in CMAs plus all those living in Census Agglomerations with populations exceeding 50,000, have mean life satisfaction 0.17 points lower than the regions in the small cities, towns, and rural areas of the rest of the country.

**Fig 6:**
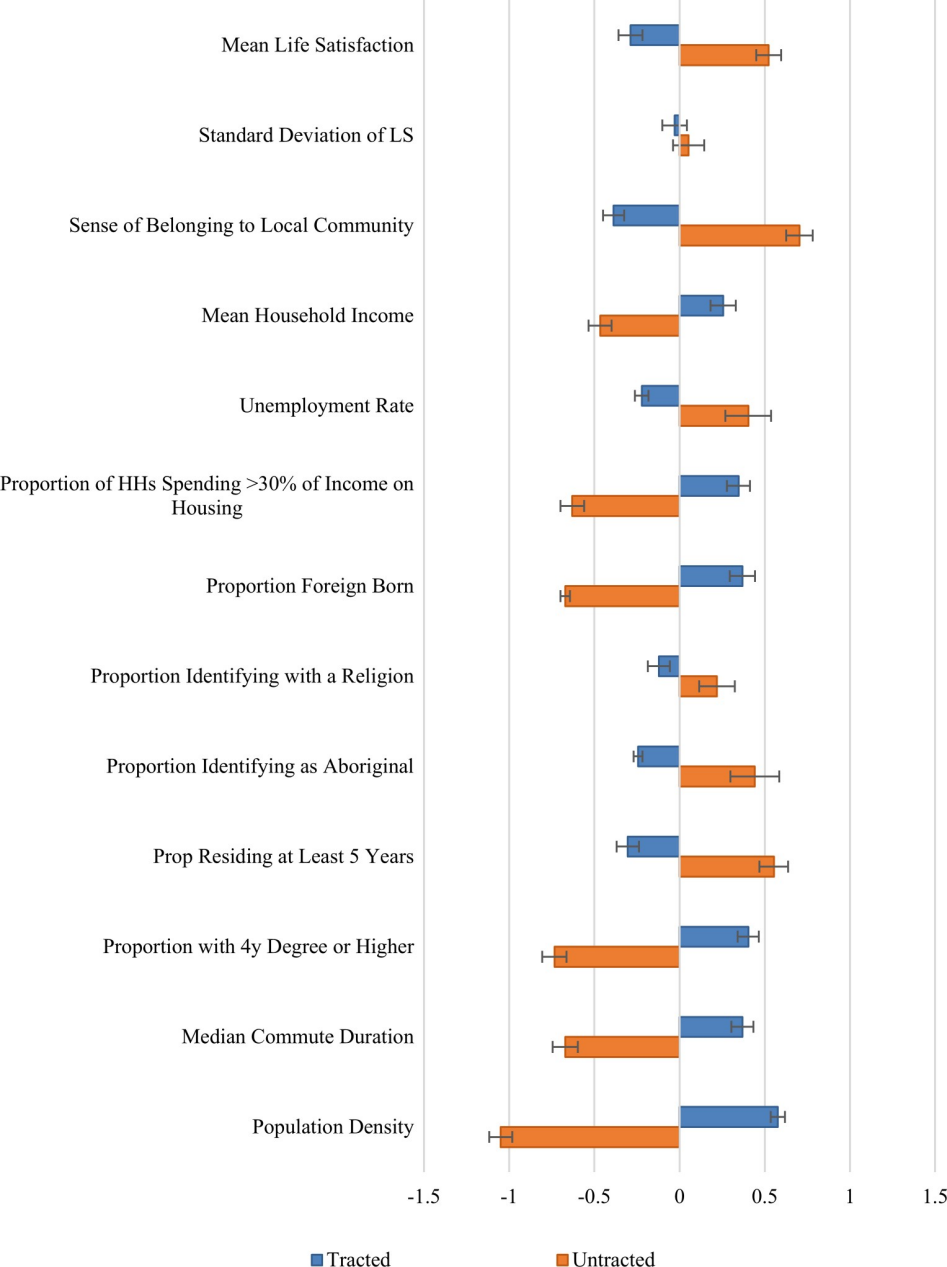
Difference of tracted and non-tracted means from the mean region. Error bars represent heteroskedasticity robust 95% confidence intervals. Values for each variable are normalized to the standard deviations given in Table 1.

The tracted and non-tracted regions are compared in Fig 6 and Table 3 in the same manner as the top and bottom quintiles, above. The average gap between urban and rural life satisfaction is about one-third as large as was found earlier between the top and bottom quintiles. Meanwhile, the sense of community belonging has an urban-rural gap almost as big as that between the top and bottom life satisfaction quintiles. Mean incomes are slightly but significantly higher in the urban areas, and unemployment rates lower. The proportion of those spending more than 30% of their incomes on housing is significantly higher in the urban areas (25% vs 18%), although the difference is slightly less than for the corresponding difference between the unhappiest and happiest quintiles (30% vs 17%). The foreign-born share of the population is also much higher in the urban areas (at 22%, compared to 6% in the rural areas), reflecting that fact that most immigrants now locate in urban areas. The fraction of the population reporting a religious affiliation is slightly but significantly higher in the rural areas (79% vs 75%), although this difference is less than between the top and bottom quintiles (82% vs 71%). The average indigenous share is also significantly higher in rural than urban areas (8% vs 3%), while it is identical in the top and bottom happiness quintiles (6.4% vs 5.3%, ns).

**Table 3:** Differences between means for urban and rural regions.

Variable	Urban	Rural	Difference
Satisfaction with Life	7.97 (0.01)	8.15 (0.01)	-0.17 (0.01)
Std. Deviation of SWL	1.65 (0.01)	1.67 (0.01)	-0.02 (0.01)
Community Belonging	0.69 (0.00)	0.78 (0.00)	-0.09 (0.00)
Log Mean HH Income	11.29 (0.01)	11.12 (0.01)	0.17 (0.01)
Unemployment (percent)	7.35 (0.08)	9.02 (0.26)	-1.67 (0.27)
Housing Over 30% of Income	0.25 (0.00)	0.18 (0.00)	0.08 (0.00)
Proportion Foreign Born	0.22 (0.01)	0.06 (0.00)	0.17 (0.01)
Proportion Religious	0.75 (0.00)	0.79 (0.01)	-0.04 (0.01)
Proportion Indigenous ID	0.03 (0.00)	0.08 (0.01)	-0.05 (0.01)
Proportion Resided 5+ Years	0.60 (0.00)	0.67 (0.00)	-0.08 (0.01)
Proportion University Degree	0.67 (0.00)	0.55 (0.00)	0.11 (0.00)
Median Commute (minutes)	21.70 (0.22)	14.71 (0.25)	6.99 (0.33)
Log Population Density	6.92 (0.06)	2.29 (0.10)	4.63 (0.12)

Heteroskedasticity robust standard errors are in parentheses.

How about the age distribution? There is a well-established U-shape in the distribution of life satisfaction scores over the life course, with life satisfaction being higher for the younger and older groups than for those in the middle [38–40]. To determine whether different age distributions could be driving the urban rural happiness gap, we aggregated Canadian NHS data for the local age distribution of the Canadian adult population into 13 age bins as used in the CCHS. These were then aggregated up to give the overall age distribution in the urban and rural parts of the country as defined in Fig 6 and Table 3. Using these relative population shares as weights, and the national average life satisfaction for each age group, we simulate the gap that would prevail on the basis of the age distribution alone. The rural and urban population distributions differ, with higher proportions of the young in the cities, of the old in rural areas, with the middle-aged shares roughly the equal in rural and urban neighbourhoods. The happy young raise the city averages, while the happy old raise the average rural scores. The net effect is very small, about 3 percent as large as the average rural/urban gap in life satisfaction, and its sign favours the rural areas, so that adjusting the average neighbourhood life satisfaction data for the effects of the differing age distributions would add slightly, although insignificantly, to the life satisfaction gap left to be explained by other factors.

Although there is no higher-education gap between the happiest and least happy communities, there is a significant difference in education levels across the urban/rural divide, with the average percentage of population with tertiary education being 67 percent in the cities vs 55 percent in the rural areas. Average commuting times are 15 minutes in the rural areas, compared to 22 minutes in the city, while population density is almost 100 times higher in the cities than in the rural areas.

**Fig 7:**
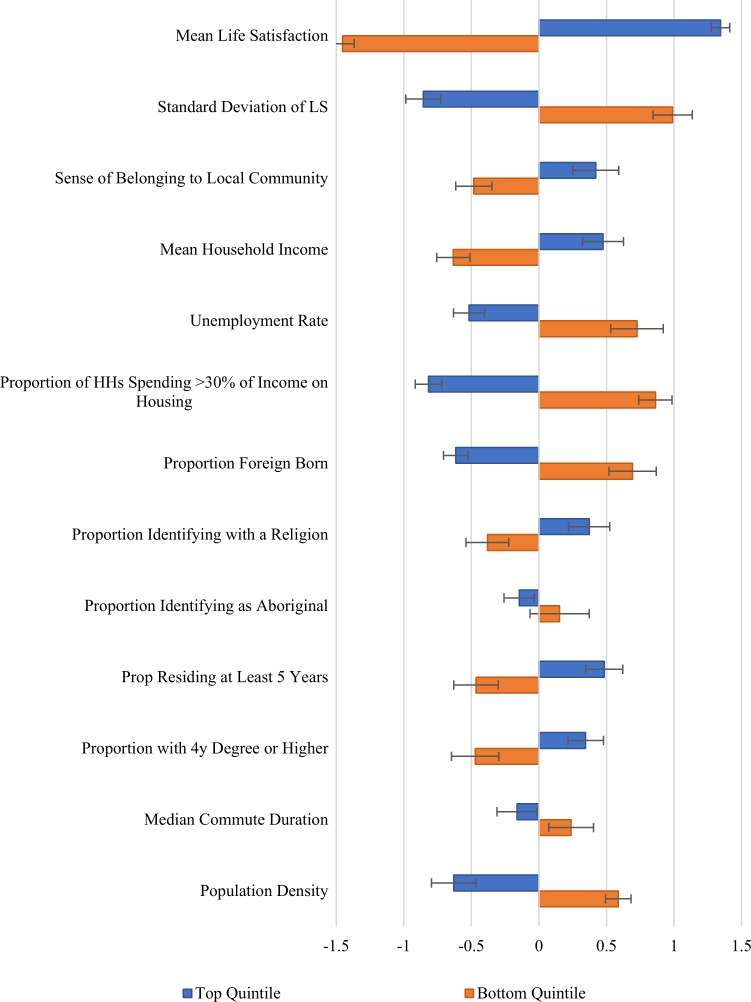
Difference of top and bottom quintile means of tracted regions from the mean tracted region. Error bars represent heteroskedasticity robust 95% confidence intervals. Values for each variable are normalized to the standard deviations given in Table 1.

**Fig 8:**
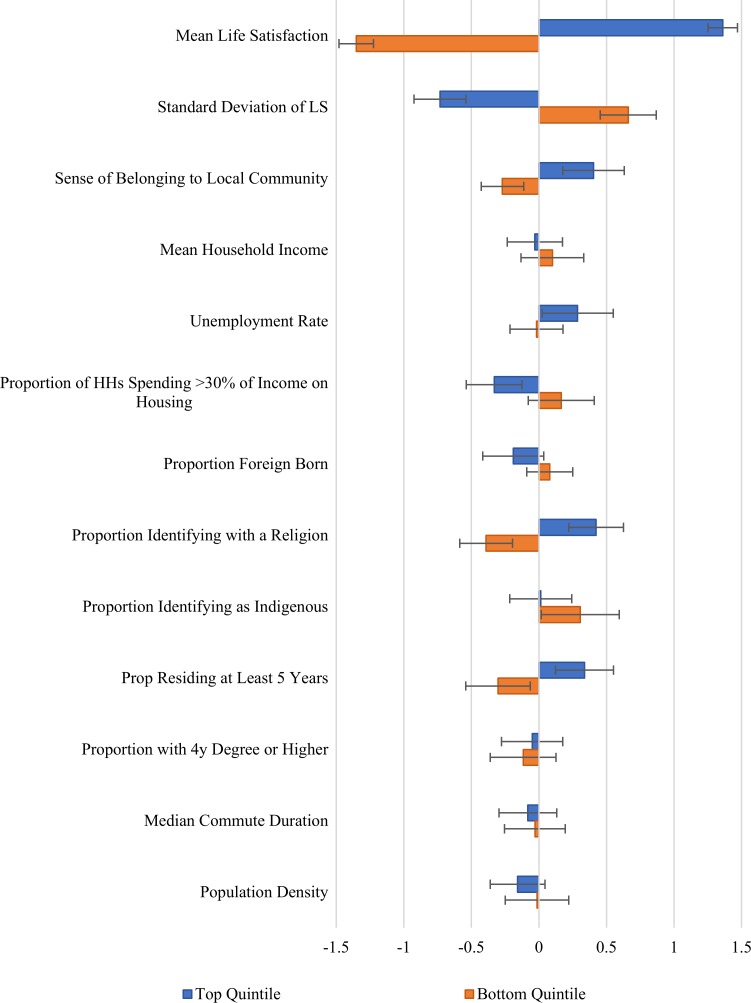
Difference of top and bottom quintile means of non-tracted regions from the mean non-tracted region. Error bars represent heteroskedasticity robust 95% confidence intervals. Values for each variable are normalized to the standard deviations given in Table 1.

We now turn, in Figs 7 and 8, which repeat Fig 5 for the urban and rural samples separately. Fig 7 examines the differences between top and bottom life satisfaction quintiles among the 776 urban communities, while Fig 8 does the same thing for the rural sample, which is slightly more than half as large. The corresponding raw means and differences are presented in Tables 4 and 5. One striking result is that even the happiest quintile of urban communities has a significantly lower average sense of community belonging than in even the least happy quintile of rural communities. Since personal connections tend to decay with distance, it might be thought that a sense of community belonging would be easier to establish where people were closer to each other, as they clearly are in urban communities. But the reverse holds true, suggesting that some features of urban life work against the maintenance of a strong sense of community belonging.

**Table 4:** Differences between means of top and bottom life satisfaction quintiles of tracted regions.

Variable	Top Quintile	Bottom Quintile	Difference
Satisfaction with Life	8.2 (0.01)	7.65 (0 .01)	0.62 (0.01)
Std. Deviation of SWL	1.48 (0.01)	1.84 (0.01)	-0.35 (0.02)
Community Belonging	0.72 (0.01)	0.66 (0.00)	0.06 (0.01)
Log Mean HH Income	11.43 (0.02)	11.12 (0.02)	0.31 (0.03)
Unemployment (percent)	6.12 (0.13)	9.04 (0.20)	-2.91 (0.24)
Housing Over 30% of Income	0.19 (0.00)	0.32 (0.00)	-0.13 (0.01)
Proportion Foreign Born	0.12 (0.01)	0.34 (0.01)	-0.22 (0.02)
Proportion Religious	0.79 (0.01)	0.70 (0.01)	0.09 (0.01)
Proportion Indigenous ID	0.03 (0.00)	0.04 (0.00)	-0.01 (0.00)
Proportion Resided 5+ Years	0.64 (0.01)	0.55 (0.01)	0.08 (0.01)
Proportion University Degree	0.70 (0.01)	0.62 (0.01)	0.07 (0.01)
Median Commute (minutes)	20.73 (0.45)	23.17 (0.50)	-2.44 (0.68)
Log Population Density	5.91 (0.14)	7.87 (0.08)	-1.97 (0.16)

Heteroskedasticity robust standard errors are in parentheses.

**Table 5:** Differences between means of top and bottom life satisfaction quintiles of non-tracted regions.

Variable	Top Quintile	Bottom Quintile	Difference
Satisfaction with Life	8.39 (0.01)	7.89 (0.01)	0.50 (0.02)
Std. Deviation of SWL	1.53 (0.02)	1.80 (0.02)	-0.27 (0.03)
Community Belonging	0.81 (0.01)	0.77 (0.01)	0.04 (0.01)
Log Mean HH Income	11.11 (0.02)	11.15 (0.02)	-0.04 (0.03)
Unemployment (percent)	10.64 (0.75)	9.28 (0.55)	1.36 (0.93)
Housing Over 30% of Income	0.16 (0.01)	0.19 (0.01)	-0.03 (0.01)
Proportion Foreign Born	0.05 (0.01)	0.06 (0.00)	-0.01 (0.01)
Proportion Religious	0.85 (0.01)	0.74 (0.01)	0.11 (0.02)
Proportion Indigenous ID	0.08 (0.02)	0.15 (0.02)	-0.07 (0.03)
Proportion Resided 5+ Years	0.71 (0.01)	0.64 (0.01)	0.06 (0.01)
Proportion University Degree	0.55 (0.01)	0.54 (0.01)	0.01 (0.01)
Median Commute (minutes)	14.48 (0.57)	13.92 (0.64)	0.56 (0.86)
Log Population Density	2.00 (0.21)	2.12 (0.29)	-0.12 (0.36)

Heteroskedasticity robust standard errors are in parentheses.

There are large life satisfaction gaps between the top and bottom quintiles in cities and in rural areas. Average life satisfaction in the top quintile of urban communities is almost as high as in the rural sample (8.27 vs 8.39, a difference that is highly significant in statistical terms). The bottom quintiles have average life satisfaction of 7.65 in the city vs 7.89, a gap twice as large as that for the top quintiles. Although the inter-quintile gaps are thus very large for life satisfaction in both city and rural areas, with something similar for well-being inequality and a sense of community belonging, the picture is quite different for most of the census-based variables. In particular, there is much more evidence of links to census variables for the urban sample than in the rural areas.

When we compare the average characteristics of the most and least happy urban communities, we find a number of large matching differences in census-based variables. In particular, in the happiest quintile of urban neighbourhoods, incomes are higher, unemployment is lower, fewer people spend more than 30% of their incomes on housing, proportions of the foreign-born are lower, religious identification is higher, education levels are higher, commuting times are shorter, and population densities are lower.

Things are very different in Fig 8 comparing lives in the top and bottom quintiles in the rural sample. There are more religious identifiers and fewer movers in the top quintile than in the bottom one. But beyond those two differences, all of the other census variables have similar averages in the top and bottom quintiles.

These correlations cannot be assumed to have causal significance at the neighbourhood level, since individual city dwellers have many neighbourhoods to choose from within the same commuting zone, and their incomes and occupations are likely to influence where they can afford to live, and where they choose to live. Indeed, the interplay between incomes, local amenities, and each individual’s choice of where to live are the basic building blocks of standard economic models of spatial equilibrium [41, 42]. Under the assumptions of costless relocation and perfect information, wages and land prices would adjust to compensate for the value of local amenities, rendering the overall welfare available in all locations equal from the perspective of a potential mover. Crucially, this result depends on the restriction that an individual must reside and work in the same location. At the level of our rural regions, which contain entire towns and small cities, homes and jobs are more likely to both be within the same community/neighbourhood, so that compensating wage differentials might help to explain why we observe less dispersion in life satisfaction levels across rural regions and fewer clear cut differences between the happiest and least happy rural regions along economic dimensions.

Consistent with this potential explanation, the correlation between the strength of the social fabric, as measured by levels of community belonging, and local income and unemployment are reversed in the urban and rural samples. Within cities, where people can live in one neighbourhood and work in another, community belonging is positively and significantly correlated with log incomes, and negatively with the local unemployment rate (0.38 and -0.09, respectively). We also find, using data from each of the nine largest CMAs, all of which have a sufficiently larger number of neighbourhoods to populate the quintiles, that average life satisfaction is significantly higher in communities that fall into the top quintile of the income distribution than those in communities the bottom quintile. We also find that average life satisfaction rises significantly moving up the quintiles for community belonging, and falls significantly for the quintiles with higher unemployment rates. This is quite different from a similar relation among rural communities, where the signs of the correlations are reversed (-0.15, and 0.36 respectively, weighted by community belonging sample size and significant at p < = 0.05 in all cases). Our ability to unpack the urban geography of cities thus adds a distinct new dimension to the nature of local differences in life satisfaction. To go further here in explaining these differences would take us too far beyond our main purpose, which is to describe our approach to dividing a nation into contiguous communities in ways that respect natural and built boundaries, thereby providing a highly granular data set with a much larger number of communities than previously available either for simple comparisons or for use as a basis for estimating neighbourhood effects.

## Conclusion

We have defined and measured the life satisfaction of 1216 Canadian neighbourhoods and communities. Our regionalization method targeted sample sizes in a range from 300–500 respondents for each of these geographic entities. This target provides an appealing trade-off between sample size and spatial resolution, as confirmed in a subsequent cross-validation exercise. We made heuristic use of road networks and natural geography to improve the extent to which our groupings can plausibly correspond to actual neighbourhoods and communities. We also ensured that all boundaries coincide with, and are generally nested within, Canadian census boundaries, so that the community-level survey information which we present can be readily combined with census-based data for the same communities as well as those at higher and lower scales.

Looking across these communities, we found a substantial range in average life satisfaction. Comparing averages in the top and bottom quintiles, life satisfaction averaged 8.33 in the happiest quintile and 7.7 in the least happy quintile. This gap of 0.6 points on the 0 to 10 scale is substantial in scale, and highly significant in statistical terms. In terms of practical significance, while it is less than 20% as large as the corresponding gap between the top and bottom quintiles of the roughly 150 countries covered by the rankings in the *World Happiness Reports*, it is half again as large as the gap previously found between Saguenay and Vancouver, the happiest and least happy CMAs, respectively, as of 2013 [13].

We then compared how lives differed in the top and bottom quintiles of our 1216 communities. Well-being equality and sense of community belonging were both significantly higher in the happy communities, while there were no significant differences in average incomes, or unemployment, or indigenous population shares. However, we did find that the top quintile communities had lower commute times, smaller shares of the population spending over 30% of their incomes on housing, smaller foreign-born population shares, and much smaller population densities, all of which are features of rural rather than urban life.

When we divided our sample into the rural and urban parts, we found life to indeed be less happy in the cities–by 0.17 points, almost half as large as the gap between the top and bottom quintiles. This was despite higher incomes, lower unemployment rates and higher education in the urban areas. On the other hand, urban dwellers were more likely to have moved recently, and less likely to have a sense of community belonging than were those in more rural areas.

What are the next steps? We are making the resulting community-level Canadian data available to other researchers, with an eye to two types of use.

First, and most readily, they provide a snapshot of variations among communities, both across and within cities, with a sample size large enough to invite examination of plausible sources of the substantial inter-community differences we have found.

Second, our data can be used, along with matching census-based data for the same geographies, as social context variables for multi-level modelling of individual-level data for life satisfaction. One natural application would be to assess the sign and size of the contextual effects from a variety of key variables. For example, what are the externalities, sometimes called ‘social multiplier’ effects [43], but more frequently ‘neighbourhood effects’ [3, 6] flowing from neighbourhood-level variation in social and economic conditions above and beyond what follows from each individual’s own circumstances? The social multiplier is often argued to be negative for incomes, based on comparison effects [23] but positive for social trust [20]. Some variables, such as inequalities in the distribution of income, health, education and life satisfaction, are defined only at neighbourhood or higher levels of aggregation and have often been argued to have negative consequences for average measures of individual well-being [44, 45]. Our method defines a large number of distinct neighbourhoods, of roughly equivalent sample size, potentially supporting better answers to these questions.
